# Mortality calculator as a possible prognostic predictor of overall survival after gastrectomy in elderly patients with gastric cancer

**DOI:** 10.1186/s12957-020-02052-x

**Published:** 2020-10-30

**Authors:** Hidenori Akaike, Yoshihiko Kawaguchi, Suguru Maruyama, Katsutoshi Shoda, Ryo Saito, Shinji Furuya, Naohiro Hosomura, Hidetake Amemiya, Hiromichi Kawaida, Makoto Sudoh, Shingo Inoue, Hiroshi Kohno, Daisuke Ichikawa

**Affiliations:** First Department of Surgery, Faculty of Medicine, University of Yamanashi, 1110 Shimokato, Chuo, Yamanashi, 4093898 Japan

**Keywords:** Gastric cancer, Elderly, Overall survival, Prognosis, Risk calculator

## Abstract

**Background:**

The number of elderly patients with gastric cancer has been increasing. Most elderly patients have associated reduced physiologic functions that can sometimes become an obstacle to safe surgical treatment. The National Clinical Database Risk Calculator, which based on a large Japanese surgical database, provides predicted mortality and morbidity in each case as the surgical-related risks. The purpose of this study was to investigate the clinical significance of the risk for operative mortality (NRC-mortality), as calculated by the National Clinical Database Risk Calculator, during long-term follow-up after gastrectomy for elderly patients with gastric cancer.

**Methods:**

We enrolled 73 patients aged ≥ 80 years and underwent gastrectomy at our institution. Their surgical risk was evaluated based on the NRC-mortality. Several clinicopathologic factors, including NRC-mortality, were selected and analyzed as the possible prognostic factors for elderly patients who have undergone gastrectomy for gastric cancer. Statistical analysis was performed using the log-rank test and Cox proportional hazard model.

**Results:**

NRC-mortality ranged from 0.5 to 10.6%, and the median value was 1.7%. Dividing the patients according to mortality, the overall survival was significantly worse in the high mortality group (≥ 1.7%, *n* = 38) than in the low mortality group (< 1.7%, *n* = 35), whereas disease-specific survival was not different between the two groups. In the Cox proportional hazard model, multivariate analysis revealed NRC-mortality, performance status, and surgical procedure as the independent prognostic factors for overall survival. For disease-specific survival, the independent prognostic factors were performance status and pathological stage but not NRC-mortality.

**Conclusion:**

The NRC-mortality might be clinically useful for predicting both surgical mortality and overall survival after gastrectomy in elderly patients with gastric cancer.

## Background

Japan has been one of the fastest aging societies in the world [1], and surgical strategies for elderly patients have been the emerging issue in geriatric medicine. Gastric cancer is the second most common cancer in Japan, in the background of an aging society, the number of elderly patients with gastric cancer has been increasing [2]. The most effective treatment for gastric cancer is radical gastrectomy with lymph node dissection [3]. However, most elderly patients have associated reduced physiologic functions that can sometimes be an obstacle to safe surgical treatments [4]. In such elderly patients, postoperative complications occur more frequently and may be occasionally fatal [4, 5]. Therefore, the indications for surgical treatment of elderly patients should be evaluated comprehensively based on a balance of radicality, safety, and appropriateness. Various studies have been performed to predict the prognosis after gastrectomy in elderly patients with gastric cancer. However, almost all studies have reported prognostic factors mainly in terms of avoiding recurrences and reviewed clinicopathologic factors in the same manner as that for nonelderly patients [6, 7].

Recently, some large-scale clinical databases have provided surgical data and surgery-related risk calculators for mortality and morbidity [8, 9]. In Japan, registration in a web-based National Clinical Database (NCD) began in 2011 and has included the clinicopathologic information, surgical results, postoperative complications, and surgery-related deaths of over 4 million cases from more than 4100 facilities over a 3-year period [10]. Information from the NCD has been considered the most reliable baseline data that reflect the common surgical practices in Japan. As a real-time feedback system on the web, the NCD Risk Calculator provides predictive risks of mortality and several main morbidities based on the registered preoperative clinical data of patients who have undergone eight major surgical procedures, including total gastrectomy [11, 12] and distal gastrectomy [13, 14].

The operative mortality calculated by the NCD Risk Calculator (NRC-mortality) has been widely used to assess the safety of surgical treatment in clinical practice. In this study, we aimed to investigate the clinical significance of NRC-mortality during the long-term follow-up after gastrectomy for elderly patients with gastric cancer, based on the assumption that the NRC-mortality would reflect the general condition of each patient.

## Methods

### Patients

Ninety-eight patients aged 80 or more years and who have undergone gastrectomy for gastric cancer at the University of Yamanashi Hospital between 2001 and 2014 were included. This study excluded 7 patients with remnant gastric cancer, 14 patients with R1 or R2 resection, and 4 patients who were lost to follow-up. Finally, 73 elderly patients were enrolled in this study, which reviewed the clinicopathologic data from the medical records. Surgical risk was evaluated based on the NRC-mortality.

Several clinicopathologic factors were selected and analyzed as predictive prognostic factors among elderly patients with gastric cancer after gastrectomy. These included patient-related factors, such as sex, performance status of Eastern Cooperative Oncology Group (ECOG-PS), Glasgow prognostic score (GPS), Onodera’s prognostic nutritional index (PNI) [15], and NRC-mortality. All these factors were calculated at the time of admission for surgery. The tumor-related factors were pathologic stage of gastric cancer (pStage), and treatment-related factors were surgical procedure, lymph node dissection, operative time, blood loss, postoperative complications defined as Clavien-Dindo grade 2 or more and adjuvant chemotherapy.

The clinicopathologic findings were classified based on the 8th edition Union for International Cancer Control TNM classification [16]. Lymph node dissection was performed in principle, according to the gastric cancer treatment guidelines of the Japanese Gastric Cancer Association [17]. Each factor was assessed as an indicator of patient prognosis, in terms of overall survival (OS) and disease-specific survival (DSS) from the date of surgery. The observation period for this study was up to 5 years after surgery.

All procedures carried out in this study were in accordance with the ethical standards of the responsible institutional and national committees on human experimentation and with the Helsinki Declaration of 1964 and its later amendments or equivalents. This study was approved by the University of Yamanashi Faculty of Medicine Ethics Committee.

### NCD Risk Calculator

The NCD Risk Calculator is one of the functions of the NCD feedback. A risk model was constructed based on the NCD registration data to calculate predictive values, such as mortality and complication rate, in patients undergoing surgery. In other words, after input of the preoperative risk of each case, the outcome was calculated from the case data registered nationwide and feedback was immediately given to each department.

**Fig. 1:**
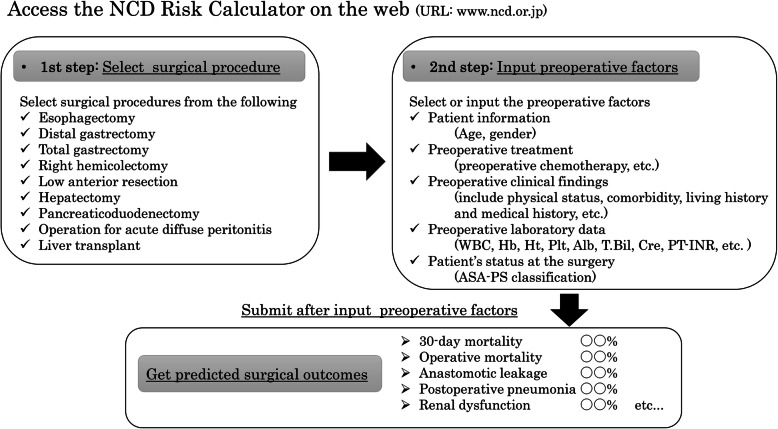
Outline of the use of the National Clinical Database risk calculator. ASA-PS: American Society of Anesthesiologists Physical Status

Using the NCD Risk Calculator is very easy. First, access the NCD Risk Calculator on the web and select the surgical procedure to perform. Next, input and submit age, sex, physical findings, comorbidities, living history, medical history, preoperative blood test values, American Society of Anesthesiologists Physical Status Classification, etc. All the items required for this entry are confirmed before surgery. The outcome of each surgery is calculated only in two steps as shown in Fig. 1. The outcome items calculated using the NCD Risk Calculator vary slightly for different surgical procedures. Common outcomes of distal gastrectomy and total gastrectomy include mortality within 30 days after surgery (30-day mortality), 30-day mortality or in-hospital mortality (up to 90 days after surgery) (operative mortality), anastomotic leakage, postoperative pneumonia, and renal dysfunction are calculated [11–14]. However, the NCD Risk Calculator is only available to NCD registered members.

### Statistical analysis

All statistical analyses were carried out using the statistical computing software R version 3.4.4 (R Foundation for Statistical Computing, Vienna, Austria). Survival curves were estimated using the Kaplan-Meier method and statistical analysis was performed using the log-rank test. The Cox proportional hazard model was used to calculate hazard ratios for OS and DSS. Statistical significance was set at *p* < 0.05.

## Results

The patient’s characteristics are shown in Table 1. Total gastrectomy was performed on 28 patients and distal gastrectomy was performed on 45 patients. Laparoscopic surgery was performed on 11 patients. Postoperative complications developed in 22 patients; the most common was pneumonia in 5 patients, followed by anastomotic leakage in 4 patients. Recurrence was observed in 15 patients, 7 of whom developed liver metastasis. During the follow-up period of 5 years after surgery, 33 patients died of gastric cancer (*n* = 13) and other diseases (*n* = 20). There were two surgery-related deaths within 90 days after surgery.

**Table 1:** Patients characteristics

	*n* = 73
Age (years)	82 [80–95]
Sex (male/female)	45/28
ECOG-PS (0/1/2/3)	16/39/17/1
Glasgow prognostic score (0/1/2)	51/16/6
Prognostic nutritional index	44.2 [31.0–59.0]
NRC-mortality (%)	1.7 [0.5–10.6]
pT (1a/1b/2/3/4a/4b)	13/29/9/8/12/2
pN (0/1/2/3a)	40/17/12/4
pStage (IA/IB/IIA/IIB/IIIA/IIIB)	32/15/5/3/12/6
Surgical procedure (total gastrectomy/distal gastrectomy)	28/45
Surgical approach (open/laparoscopic)	62/11
Lymphadenectomy (<D2/D2)	49/24
Operative time (min)	241 [88–800]
Blood loss (ml)	351 [28–3257]
Post-operative complications^a^ (yes/no)	22/51
Adjuvant chemotherapy^b^ (yes/no)	8/15

*ECOG-PS* Eastern Cooperative Oncology Group Performance Status, *NRC-mortality* The operative mortality calculated by the National Clinical Database Risk Calculator

^a^ Clavien-Dindo grade 2 or more

^b^ Patients with pStage II or III except for T1

**Fig. 2:**
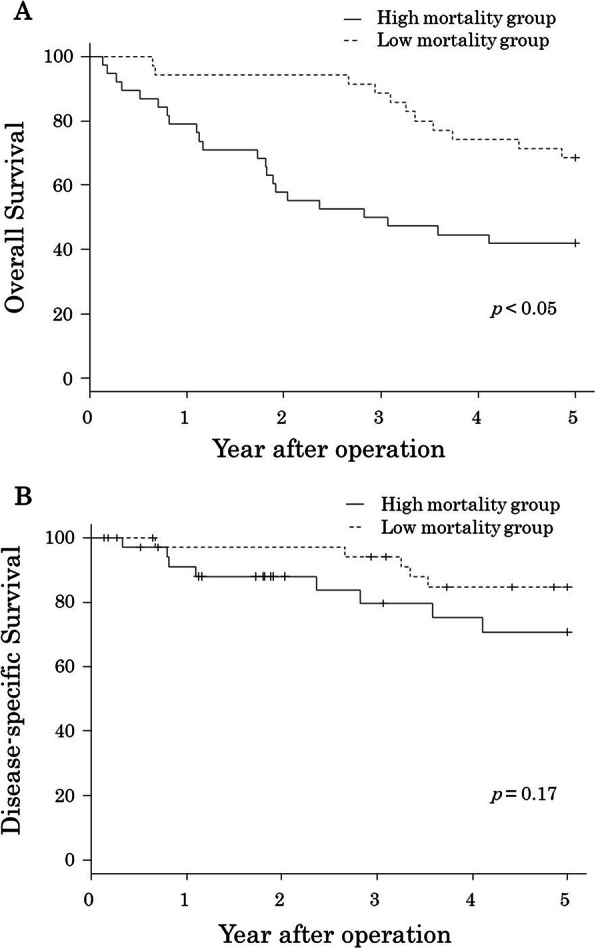
Survival curves of the group of elderly patients with gastric cancer after gastrectomy, according to the operative mortality risk calculated by the National Clinical Database risk calculator. **a** Overall survival. **b** Disease-specific survival

NRC-mortality ranged 0.5–10.6% (median, 1.7%). The patients were divided into two groups according to the median value, high mortality group (NRC-mortality, ≥ 1.7%) and low mortality group (NRC-mortality, < 1.7%), to investigate the postoperative prognostic usefulness of the NRC-mortality. Thirty-eight patients were classified as the high mortality group, whereas 35 patients were classified as the low mortality group. Figure 2 shows the survival curves of each group. Compared with the low mortality group, high mortality group had significantly worse prognosis in terms of OS, but there was no difference in DSS. No significant difference was noted in the rates of patients with pStage II or III except for T1 who received adjuvant chemotherapy between the high- and low-mortality groups. In addition, no difference was also observed between the two groups in the rates of patients who received chemotherapy after recurrence.

In the Cox proportional hazard model, univariate analysis showed that NRC-mortality, ECOG-PS, PNI, surgical procedure, and postoperative complications were significantly correlated with OS, however, sex, GPS, pStage, lymphadenectomy, operative time, blood loss, and adjuvant chemotherapy were not. Multivariate analysis showed that ECOG-PS, NRC-mortality, and surgical procedure were the independent prognostic factors for OS (Table 2). By contrast, ECOG-PS, NRC-mortality, and pStage were significantly correlated with DSS in the univariate analysis. In multivariate analysis, the independent prognostic factors for DSS were ECOG-PS and pStage and not NRC-mortality (Table 3).

**Table 2:** Survival analysis of variables predicting prognostic factors for overall survival in elderly patients with gastric cancer after gastrectomy

	Univariate			Multivariate		
Variable	HR	95%CI	*p* value	HR	95%CI	*p* value
Sex (male)	1.613	0.768–3.391	0.207			
ECOG PS (≧ 2)	4.727	2.356–9.485	< 0.001	2.564	0.163–0.932	0.034
Glasgow prognostic score (≧ 1)	1.599	0.786–3.255	0.195			
Prognostic nutritional index	0.938	0.888–0.991	0.023	0.980	0.919–1.045	0.541
NRC-mortality	1.336	1.195–1.494	< 0.001	1.213	1.053–1.396	0.007
pStage (≧ II)	1.976	0.994–3.926	0.052			
Surgical procedure (total gastrectomy)	2.509	1.259–4.997	0.009	2.285	1.115–4.682	0.024
Lymphadenectomy (< D2)	1.414	0.657–3.043	0.376			
Operative time	1	0.996–1.003	0.734			
Blood loss	1	0.999–1.001	0.997			
Postoperative complications^a^ (yes)	2.034	1.010–4.093	0.047	1.396	0.653–2.983	0.390
Adjuvant chemotherapy (yes)	0.476	0.641–6.890	0.220			

*ECOG PS* Eastern Cooperative Oncology Group Performance Status, *NRC-mortality* The operative mortality calculated by the National Clinical Database Risk Calculator, *HR* hazard ratio

^a^ Clavien-Dindo grade 2 or more

**Table 3:** Survival analysis of variables predicting prognostic factors for disease-specific survival in elderly patients with gastric cancer after gastrectomy

	Univariate			Multivariate		
Variable	HR	95%CI	*p* value	HR	95%CI	*p* value
Sex (male)	0.831	0.279–2.473	0.739			
ECOG PS (≧ 2)	3.73	1.203–11.57	0.023	7.978	1.633–38.97	0.01
Glasgow prognostic score (≧ 1)	2.428	0.815–7.24	0.111			
Prognostic nutritional index	0.927	0.850–1.012	0.089			
NRC-mortality	1.272	1.039–1.558	0.020	1.038	0.794–1.357	0.785
pStage (≧ II)	5.464	1.679–17.78	0.005	9.375	2.202–39.92	0.002
Surgical procedure (total gastrectomy)	1.865	0.623–5.583	0.265			
Lymphadenectomy (< D2)	0.623	0.209–1.854	0.395			
Operative time	1	0.991–1.003	0.324			
Blood loss	1	0.998–1.001	0.696			
Postoperative complications^a^ (yes)	2.744	0.921–8.178	0.070			
Adjuvant chemotherapy (yes)	0.386	0.337–19.94	0.360			

*ECOG PS* Eastern Cooperative Oncology Group Performance Status, *NRC-mortality* The operative mortality calculated by the National Clinical Database Risk Calculator, *HR* hazard ratio

^a^ Clavien-Dindo grade 2 or more

## Discussion

Surgical resection for elderly patients is accompanied with significant perioperative mortality and frequent postoperative complications [4, 5]. In addition, the life expectancy of this population is limited, in comparison with that of young patients. Therefore, the indications of surgical treatment should be discussed comprehensively based on various patient- and tumor-related factors. In this study, 20 patients (27%) died from diseases other than gastric cancer during the follow-up period of 5 years after gastrectomy. This was an indispensable result, and OS, including deaths from diseases other than gastric cancer, should be considered equally important as DSS in elderly patients. Surgical indications should be determined by considering not only DSS but also OS, especially for elderly patients. The results of the current study clearly showed that NRC mortality is useful for predicting OS but not DSS.

Previous reports have identified sex [18, 19]. ECOG-PS [19], surgical procedure [4, 7], PNI [7, 20], and postoperative complications [7, 19], as predictors of OS among elderly patients with gastric cancer after gastrectomy. In our study, NRC-mortality, in addition to ECOG-PS and surgical procedure, was found to be the independent predictors of OS. When concerning surgical procedures, total gastrectomy may sometimes result in malnutrition [21] and aspiration pneumonia, particularly in elderly patients [22]. Given these insights, it may be better to avoid total gastrectomy for elderly patients with gastric cancer. On the other hand, postoperative complications have been recognized as reliable prognostic factors after curative resection of various cancers, including gastric cancer [7, 17, 23–25]. Based on our recent analysis that demonstrated a possible correlation between the adverse prognostic effect and immune status of patients [26], the immunocompromised state of elderly patients might have affected our current findings.

The NCD Risk Calculator was originally a tool for the prediction surgical morbidity and short-term surgical mortality [10]. The NRC-mortality was calculated based on logistic analysis from a large-scale NCD database, in which surgery-related deaths were registered from various causes, including deaths secondary to complications, worsening of comorbidities, and sudden deaths of unknown cause. In this study, we found that the NRC-mortality predicted not only the short-term mortality rate but also the long-term outcomes of elderly patients with gastric cancer after gastrectomy. In this population, the perioperative mortality risk might correlate with the risks of death secondary to worsening of comorbidities and of sudden deaths in both the perioperative and late phases after gastrectomy and, consequently, might be related with OS, as shown by our analysis. Furthermore, no elderly patients with NRC-mortality > 4.1 survived for 5 years postoperatively, which may indicate that the higher the NRC-mortality, the lower the expected survival.

POSSUM (Physiological and Operative Severity Score for the enUmeration of Mortality and morbidity) [27] and E-PASS (Estimation of Physiologic Ability and Surgical Stress) [28] are well-known prognostic scoring systems that are based on statistical analysis of actual surgical data. However, both scoring systems provide a comprehensive prognostic assessment only after surgery and may not be suitable for accurately predicting the surgical risk prior to surgery. Moreover, the E-PASS scoring system shows that postoperative complications mainly depend on the surgical stress score, which is the actual surgical invasive outcome score [28]. Given this, the NRC-mortality rate, compared with POSSUM and E-PASS, may be more ideal tool for perioperative risk assessment of the short- and long-term outcomes. Risk assessment based on the POSSUM scoring system is known to deviate from the actual results of a limited population, such as low-risk cases and elderly patients [29, 30]. Moreover, assessments using these scoring systems have been reported to not reflect the prognosis of elderly patients [18].

There were some limitations in this study. First, it was small-scale and was done at a single institution. Further multi-institutional analyses of large number of patients should be conducted to confirm the current results. Second, the cohort included only a small number of patients who underwent laparoscopic gastrectomy, which is less invasive and may affect the long-term outcome in elderly patients. Concerning laparoscopic and robotic procedures for the elderly, huge numbers of patients’ clinicopathological information would be collected in the NCD system, which shall elucidate the clinical utility of the NRC-mortality in minimally invasive surgeries in the future.

In conclusion, the NRC-mortality, as well as ECOG-PS and surgical procedure, might be clinical useful for predicting not only surgical mortality but also OS after gastrectomy in elderly patients with gastric cancer. As much as possible, gastrointestinal surgeons should avoid performing total gastrectomy on elderly patients with gastric cancer and consider treatment without gastrectomy, particularly in cases with poor PS. The NCD Risk Calculator may help in the proper surgical risk assessment of such patients.

## Data Availability

This was not applicable to this manuscript.

## Abbreviations

NCD: National Clinical Database
NRC-mortality: Operative mortality calculated by the National Clinical Database Risk Calculator
ECOG-PS: Performance status of the Eastern Cooperative Oncology Group
GPS: Glasgow prognostic score
PNI: Onodera’s prognostic nutritional index
pStage: Pathologic stage of gastric cancer
OS: Overall survival
DSS: Disease-specific survival
